# Fungal names: a comprehensive nomenclatural repository and knowledge base for fungal taxonomy

**DOI:** 10.1093/nar/gkac926

**Published:** 2022-10-22

**Authors:** Fang Wang, Ke Wang, Lei Cai, Mingjun Zhao, Paul M Kirk, Guomei Fan, Qinglan Sun, Bo Li, Shuai Wang, Zhengfei Yu, Dong Han, Juncai Ma, Linhuan Wu, Yijian Yao

**Affiliations:** Microbial Resource and Big Data Center, Institute of Microbiology, Chinese Academy of Sciences, Beijing 100101, China; Chinese National Microbiology Data Center (NMDC), Beijing 100101, China; State Key Laboratory of Mycology, Institute of Microbiology, Chinese Academy of Sciences, Beijing 100101, China; State Key Laboratory of Mycology, Institute of Microbiology, Chinese Academy of Sciences, Beijing 100101, China; State Key Laboratory of Mycology, Institute of Microbiology, Chinese Academy of Sciences, Beijing 100101, China; Jodrell Laboratory, Royal Botanical Gardens, Kew, Richmond, Surrey TW9 3DS, UK; Microbial Resource and Big Data Center, Institute of Microbiology, Chinese Academy of Sciences, Beijing 100101, China; Chinese National Microbiology Data Center (NMDC), Beijing 100101, China; Microbial Resource and Big Data Center, Institute of Microbiology, Chinese Academy of Sciences, Beijing 100101, China; Chinese National Microbiology Data Center (NMDC), Beijing 100101, China; Microbial Resource and Big Data Center, Institute of Microbiology, Chinese Academy of Sciences, Beijing 100101, China; Chinese National Microbiology Data Center (NMDC), Beijing 100101, China; Microbial Resource and Big Data Center, Institute of Microbiology, Chinese Academy of Sciences, Beijing 100101, China; Chinese National Microbiology Data Center (NMDC), Beijing 100101, China; Microbial Resource and Big Data Center, Institute of Microbiology, Chinese Academy of Sciences, Beijing 100101, China; Chinese National Microbiology Data Center (NMDC), Beijing 100101, China; Microbial Resource and Big Data Center, Institute of Microbiology, Chinese Academy of Sciences, Beijing 100101, China; Chinese National Microbiology Data Center (NMDC), Beijing 100101, China; Microbial Resource and Big Data Center, Institute of Microbiology, Chinese Academy of Sciences, Beijing 100101, China; Chinese National Microbiology Data Center (NMDC), Beijing 100101, China; Microbial Resource and Big Data Center, Institute of Microbiology, Chinese Academy of Sciences, Beijing 100101, China; Chinese National Microbiology Data Center (NMDC), Beijing 100101, China; State Key Laboratory of Mycology, Institute of Microbiology, Chinese Academy of Sciences, Beijing 100101, China

## Abstract

Fungal taxonomy is a complex and rapidly changing subject, which makes proper naming of fungi challenging for taxonomists. A registration platform with a standardized and information-integrated database is a powerful tool for efficient research on fungal taxonomy. Fungal Names (FN, https://nmdc.cn/fungalnames/; launched in 2011) is one of the three official fungal nomenclatural repositories authorized by the International Nomenclature Committee for Fungi (NCF). Currently, FN includes >567 000 taxon names from >10 000 related journals and books published since 1596 and covers >147 000 collection records of type specimens/illustrations from >5000 preserving agencies. FN is also a knowledge base that integrates nomenclature information with specimens, culture collections and herbaria/fungaria, publications and taxonomists, and represents a summary of the history and recent advances in fungal taxonomy. Published fungal names are categorized based on well-accepted nomenclature rules and can be readily searched with different keywords and strategies. In combination with a standardized name checking tool and a sequence alignment-based identification package, FN makes the registration and typification of nomenclatural novelties of fungi convenient and accurate.

## INTRODUCTION

‘Fungi’ is a non-taxonomic term (usually indicated without a capital and not italicized) that encompasses several unrelated groups traditionally studied by mycologists—the kingdom of the ‘true’ *Fungi*, the *Oomycota* and several other fungus-like organisms (1). A total of >150 000 species have been described, but the estimated total number of fungal species may be as high as 2.2–3.8 million (1), thus posing a great challenge to describe new fungal species in terms of both taxonomy and nomenclature. Nomenclature of *Fungi* and fungus-like organisms is currently regulated by the International Code of Nomenclature for algae, fungi, and plants (referred to as the Code below) and updated every 6 years (2) and amended by the Nomenclature Session of the International Mycological Congress (IMC) under the auspices of the International Mycological Association (IMA), held every 4 years (2,3). The updated Code versions are referred to in relation to the location of the International Botanical Congresses (IBC) that ratified them, e.g. the 18th Congress in Melbourne (2011) and the 19th Congress in Shenzhen (2017). Despite the improvements in information sharing brought about by the internet, the existing literature contains a plethora of synonyms, isonyms, homonyms, orthographic variants and misapplied names that are not in accordance with the standard nomenclature system (4). Accordingly, collection and standardization of current and novel fungal taxa are of great importance.

Since the introduction of the Melbourne Code, new fungal names (names of new taxa, new combinations, replacement names, and names at new ranks) published on or after 1 January 2013 must, in the formal description (or protologue), cite the identifier issued for the name by a recognized repository (5) prior to publication. The subsequent Shenzhen Code stipulated that the designation of a fungal lectotype, neotype, or epitype of fungi also must cite an identifier on or after 1 January 2019 (2). Fungal Names (FN) was established in September 2011, and recognized as one of the three global fungal name registration repositories in December 2012 by the Nomenclature Committee for Fungi (NCF) and ratified by the IMA. The other two repositories are Index Fungorum (IF, http://indexfungorum.org/Index.htm) and MycoBank (MB, https://www.mycobank.org/). According to the Code, any nomenclatural novelty of fungi must obtain an identifier from one of these three repositories and cite it in the protologue of the publication before being considered to be valid. The nomenclatural novelties and identifiers are mutually accepted and shared among the three repositories (current registration identifier allocations: IF 550000–569999, FN 570000–579999, MB 800000–899999): their websites are synchronized upon monthly updates via API or download links.

Taxon names are key to link various databases that store information on different aspects of the organisms, particularly in the era of big data. Taxonomists and name users rely on taxon names to acquire information on the organism of interest, including scientific names, (type) specimens, preserving agency(-ies) and formal abbreviation, journals for effective publication, and the experts in the field from various websites. FN is also a knowledge base that addresses such needs by integrating nomenclature information with specimens, culture collections and herbaria/fungaria, publications and taxonomists. FN is a comprehensive collection of information on the development of fungal taxonomy from a historical timeline spanning 400 years on a global scale. Based on fungal nomenclature rules, the historically published fungal names are categorized into a standardized classification system and thus can be readily searched using a variety of search terms. In combination with a standardized name-checking tool and a sequence alignment-based identification package, FN provides a standardized and information-integrated database with a registration platform for efficient research on fungal taxonomy.

## FUNCTIONS AND USER INTERFACE

**Figure 1.**
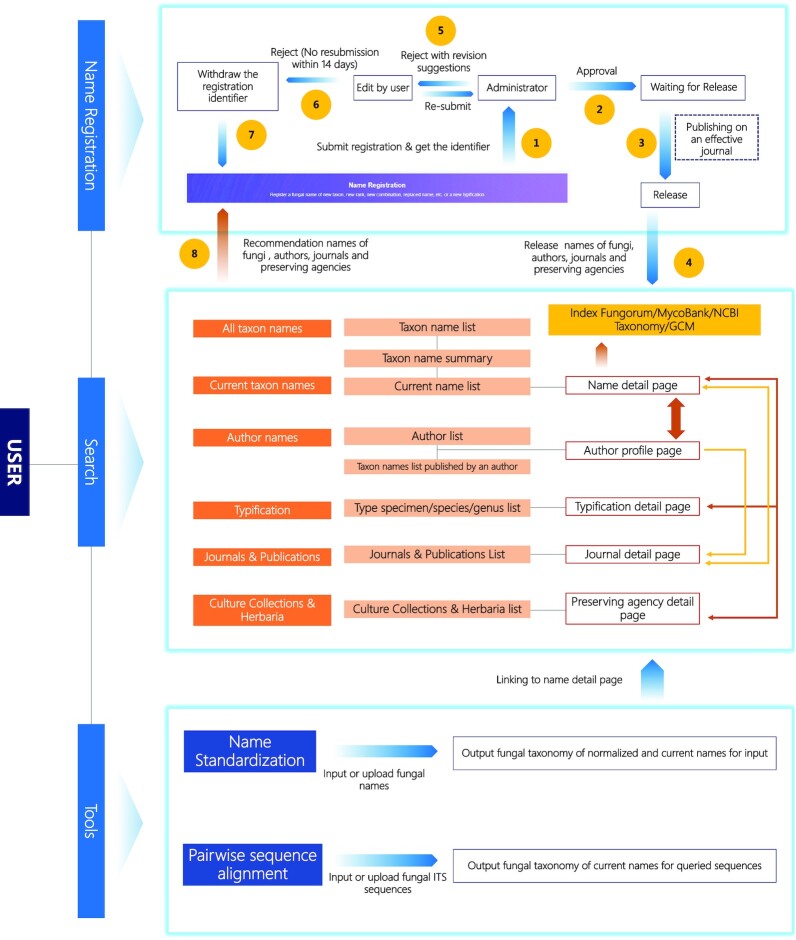
User guide of FN. An identifier for nomenclature novelties is issued immediately after the submission of the registration (Step 1) and administrative approval (Step 2–4). The identifier will be withdrawn if no reply to the ‘Revision Suggestion’ is received within 14 days (Step 5–7). Search functions are provided for all taxon names, current names, author names, typification, journals & other publications and culture collections and herbaria. For each query, a name list with an interconnecting detail page for each name is returned. Useful tools for normalizing taxon names or preliminary identification of fungal internal transcribed spacer (ITS) sequences are also provided by FN. All taxon names in the result are linked to detail pages in FN.

FN is a one-stop platform for fungal name registration, typification, name status search, standard name curation and interactive statistics (Figure 1).

### Name registration

FN provides the functions of registration, editing and release of a new taxon, new combination, new name and new typification. After registration, a unique identifier of the name/type that can be cited in the publication is issued. The identifier issued by any of the three repositories (FN, MB, IF) is unique and mutually recognized. Upon multiple applications for a fungal name/type by the authors, only one identifier will be used in the publication; the others will become invalid when the name is published. In addition to the 27 principal and secondary ranks of taxa, FN features ‘other rank’ to accommodate unforeseeable scenarios where changes to the current taxonomy are indicated (6). FN prevents a nomenclature novelty from becoming a homonym by comparing it with a standard taxon name database and normalize the input information by offering options of existing authors of fungal taxa, agencies preserving specimens, and collection countries. By using this feature, taxonomists no longer need to check the pre-published names based on the basic rules of the Code. To expedite the registration process, FN allows for conditional pre-approval immediately after the completion of the registration form. An administrator will check the registration within 24 hours and give feedback (APPROVAL, REJECTED or REVISION SUGGESTION). The pre-issued identifier will be withdrawn if there is no response or revision to the registration information within 14 days after REVISION SUGGESTION.

### Multiple search functions and display pages

**Figure 2.**
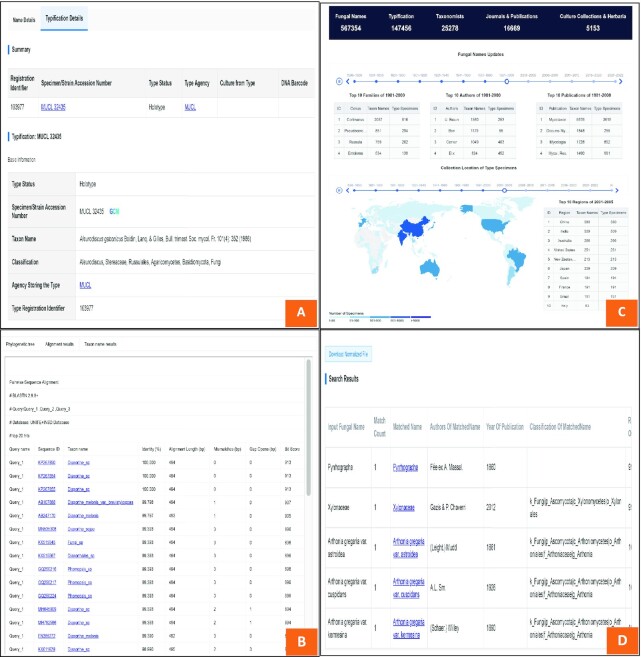
Detail pages of names of taxon (author/preserving agency/journal) (**A**), identification (**B**), statistics (**C**) and name standardization (**D**). (A) Detail page of taxon name shows the information of fungal taxonomy, typification and bibliography, and links to detail pages of author, preserving agency and journal. (B) Identification result of Pairwise Sequence Alignment for ITS sequence(s). The result supplies phylogenetic tree, alignment result, taxon name result with links to NCBI (Sequence ID) and names in FN (Taxon name). (C) Statistics page shows the summary of main data in FN. (D) Result of Name Standardization. This tool helps to normalize the fungal names with misspellings to standardized format in FN and provides taxonomy information of normalized names and corresponding current names.

FN integrates information from publications and other important fungal taxonomy databases to reduce unnecessary switches between different platforms. A quick taxonomic overview of a certain taxon from different aspects is available upon searching a specific rank (infraspecific taxa, species, genus, higher ranks), epithet, year of publication or the registration identifier of a taxon name. The profile of an interested taxonomist is also available upon search using the taxonomist's name. All the indexed names of taxon/author/agency/journal have their own pages containing detailed information (Figure 2). These pages are interconnected by taxon names. If a name is available in the other two recognized repositories (IF and MB) or NCBI Taxonomy (7), external links are provided.

### Identification and search by marker genes

DNA barcoding is a powerful tool for the rapid identification of fungal specimens (8), and the fungal barcode locus within the internal transcribed spacer (ITS) region is recommended by a large group of authors in collaboration with the Consortium for the Barcode of Life (CBOL) (9). FN provides a tool for pairwise sequence alignment based on the fungal ITS sequence dataset from the UNITE (https://unite.ut.ee) for preliminary identification of fungi. Fungal name links in FN are provided with hit results.

### Statistics

Real time statistics of current names displayed on the homepage provide an overview of the updated status of principal ranks of taxa in *Fungi* and fungus-like organisms. The former is based on current names in 20 phyla of Kingdom *Fungi*: *Aphelidiomycota* (10), *Basidiobolomycota* (11), *Blastocladiomycota* (12), *Calcarisporiellomycota* (10), *Chytridiomycota* (11), *Entomophthoromycota* (13), *Glomeromycota* (14), *Kickxellomycota* (10), *Monoblepharomycota* (11), *Mortierellomycota* (10), *Mucoromycota* (11), *Neocallimastigomycota* (15), *Olpidiomycota* (16), *Sanchytriomycota* (17), *Zoopagomycota* (18), *Caulochytriomycota* (19), *Rozellomycota* (20), *Ascomycota* (21), *Basidiomycota* (22) and *Entorrhizomycota* (23). Fungus-like taxon names are based on the current names in phyla of *Oomycota* (24) and *Mycetozoa* (25) and taxa of *Labyrinthulea* (also known as *Labyrinthulomycetes*)*, Plasmodiophorida* and *Acrasida* (24). These taxon groups are updated when more information becomes available in the literature.

FN provides an interactive dashboard with a statistical overview of the most important data fields. The validly published taxon names with the largest numbers were calculated by the years. The collection locations, deposited culture collections and herbaria/fungaria of type specimens are also displayed.

### Name standardization tools

‘One fungus one name’ is the highest standard for fungal nomenclature. Some of the taxon names in the existing literatures or databases may be synonyms, variants or invalidly published names due to a variety of reasons. This may lead to inaccurate search results. For example, *Abacina amphibia* is a synonym of *Rhizocarpon amphibium*; if all synonyms were treated as different species, the richness or diversity of the community should deviate from the truth. In order to help users to get unique current taxon name for one species, FN provides a tool for name standardization that can rapidly convert any input fungal name to its currently used formal name with corresponding taxonomic status (if available). The tool compares the input name with all existing synonyms, homonyms and orthographic variants and provides a list of the year, author, and registration identifier information of all matched items for selection by the users. If there is uncertainty about the exact name of the taxon or typographic errors, users may choose ‘fuzzy search’ to list all similar names to the input with the individual percentage of identity. The ‘fuzzy search’ will find the most similar currently used fungal name based on the names the user provided.

## DATA SOURCE AND PROCESSING

### Fungal names database

FN includes >567 000 taxon names from over 10 000 genera and 149 000 species, covering fungi, fossil fungi and organisms that were once regarded as fungi. More than 120 000 collection records of specimens and 4000 records of illustrations are holotype, lectotype, epitype or neotype of species or infraspecific taxa. The historic taxon name data are mostly shared by Index Fungorum. The data includes information from >100 data fields (e.g. name of taxon, classification, authors, year of publication, references and registration identifier). Data exchange of the newly released fungal names among the three recognized repositories occurs monthly, and includes novel taxon names, updates of existing names, information on type material, publications, classification as well as new name registrations and collection records. FN provides application programming interface (API, https://nmdc.cn/fungalnames/towebservice) for data exchanging and data sharing.

**Figure 3.**
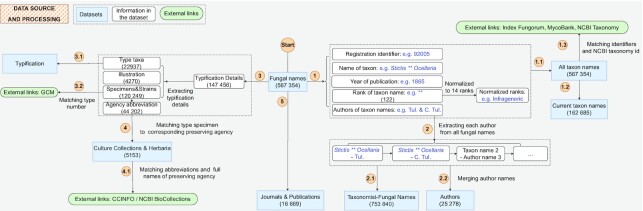
Data source and processing of FN. (1) All taxon names are cleaned and standardized to constitute the datasets of *All Taxon Names* and *Current Taxon Names* (1.1, 1.2). Taxon names are matched with and linked to names in Index Fungorum, MycoBank and NCBI Taxonomy if available (1.3). (2) Datasets of *Authors* and *Taxonomist-Fungal Names*: Authors’ names re separated and extracted from each taxon names to constitute the one author one fungal name dataset (2.1). Author names with duplicates are merged based on the information of author names, year of publication of taxon name and average publication interval of taxonomists (2.2). (3) Typification details of each taxon name, if available, are extracted and separated into three type status (type specimen, type species and type genus/phylum) and constitute the *Typification* dataset (3.1). Type specimens are linked to more details in Global Catalogue of Microorganisms (GCM) via strain numbers and taxon names (3.2). (4) Preserving agencies abbreviation are separated from type specimens and matched with dataset of Culture Collection & Herbaria. External information about the preserving agencies in Culture Collections Information Worldwide (CCINFO) and NCBI are matched via agency abbreviations and full names (4.1). (5) Journals or books that have published fungal names or are relevant to fungal taxonomy constitute the *Journal & Publication dataset*.

To improve data quality and serve a variety of functions, data are subjected to a series of processing that includes cleaning, reclassification, crosslinking and analyzing (Figure 3). First, the names are thoroughly verified and validated, and the 122 existing rank-denoting terms of taxa are reclassified to 14 standard principal and secondary ranks (seven principal ranks of taxa: kingdom, phylum, class, order, family, genus, species and seven corresponding secondary ranks—all ranks under a specific principal rank).

According to the Code, the type (e.g. holotype, lectotype, epitype or neotype) serves as an essential link between a physical element and a name. The type of a species or infraspecific taxon (e.g. a subspecies, variety or forma) can be either a specimen or strain conserved in a biorepository such as a fungarium, culture collection or an illustration under some circumstances. The type specimen number, type status and the abbreviation of biorepository are extracted from publications. FN collects and integrates the commonly accepted culture collections and herbaria/fungaria recommended by the Code from publicly available resources (Index Herbariorum, http://sweetgum.nybg.org/science/ih/) (26,27). As of June 2022, over 800 culture collections and about 4300 herbaria/fungaria and museums have been integrated into the culture collections and herbaria dataset. Abbreviations of preserving agencies in FN are standardized, and 44 202 specimens from 120 249 collection records are mapped to biorepository.

The Taxonomist-Fungal Name dataset includes 753 840 combined author-taxon name cases retrieved from authors in each fungal name (e.g. *Linocarpon bambusicola*—L. Cai and *Linocarpon bambusicola*—K.D. Hyde extracted from *Linocarpon bambusicola* L. Cai & K.D. Hyde). Authors were considered identical if the spelling of their two names are identical and the year of publication fall into the average publication interval of the taxonomist. The author dataset includes a total of 25 278 authors corresponding to the 753 840 combined taxonomist-fungal name cases with detailed profiles, and 3457 authors with comprehensive details (including their standard abbreviated name, full name, date of birth or death, the first published fungal name and year of publication). To help taxonomists publish new discoveries in authoritative journals and increase access by the public, FN links to >10 000 mycology related journals or books, with the numbers of fungal names and years of publication.

## STATISTICS AND DISCUSSION

FN maintains a record of mycological names that spans over 400 years (Table 1). Among 567 354 records of taxon names, 535 450 (94.4%) records are species or infraspecific taxa, and the remaining records are names at higher ranks (e.g. family, order, class, phylum, kingdom and their secondary ranks), including 338 955 (60.0%) names originally reported as new taxa (310 433 at the rank of species or below), 182 289 (32.1%) combinations, 7735 (1.4%) new names (replacement name or nomen novum), 35 234 (6.2%) orthographic variants and 3141 (0.6%) that are uncertain (the name type cannot be defined according to the data available).

**Table 1.** Information of taxon names at seven principal ranks and infraspecific rank

Rank	All taxon names*	Fungal current names^†^	Author	Typification of all taxon names	Journal with taxon names	Country & region	Location
Kingdom	12	3		3641			
phylum	98	20					
class	260	67					
order	608	284					
family	2996	1077					
genus	20450	10865		19296			
species	421510	149507		124519			
infraspecifics	113940						
Total	559874	161823	25278	147456	9037	224	170161

*All taxon names: including names of fungi (s.l.) and organisms once have been recognized as fungi and only taxon names in main ranks and infraspecific ranks are included in the statistics. ^†^Fungal current names: only higher taxa of fungi (s.l.) at main ranks with currently used genus names are included.

**Figure 4.**
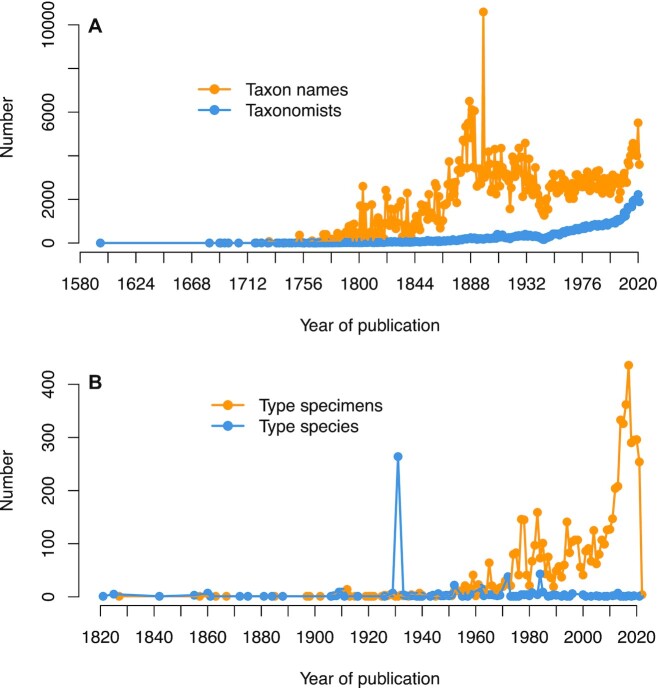
The annual increase of published taxon name and type specimens/species. (**A**) Number of taxonomists (blue) and taxon names (orange) published each year. 1596–1869 ascending phase, 1870–1899 steep phase, 1900–2009 constant phase, 2010–present second steep phage. The sharp increase in 1882–1898 coincided with the publication of *Revisio generum plantarum*, *Sylloge fungorum* and *Enchiridion Fungorum*, in *Europa Media Præsertim* in *Gallia vigentium*. (**B**) Designation of type species (blue) and type specimens (specimens and illustrations, orange) each year.

The number of fungal names continue to increase over time (Figure 4A). From the publication of the first species of fungi in 1596 (Figure 4) to 1869, fungal names increased at a rate comparable to the increase of taxonomists (ascending phase). From 1870 to 1899, the annual increase of both fungal names and taxonomists was rapid, likely due to the increasing use of microscopes and improved sampling efforts (steep phase) (28). The increasing trend of both fungal names and taxonomists was reversed in the first half of the 20th century, apparently due to the two World Wars. During the second half of the 20th century, the number of newly published fungal names remained stable. The late 20th century witnessed a sharp increase in the numbers of published fungal names and taxonomists, correlating with the introduction of molecular methods and the DNA barcodes (29,30). In the 21st century, globalization promoted the cooperation between taxonomists (internationally, inter-institutionally and inter-disciplinarily) (1) and accelerated the development of fungal taxonomy. Based on the history of fungal taxonomy, a peaceful environment and an adequate number of scientists are clearly mandatory for the advancement in this field. Breakthroughs in technologies and cooperation among scientists are also critically important.

Designation of a nomenclatural type is of paramount importance since it provides a scientifically solid reference for further research (2,3). Type designation consists of multiple levels: from infraspecific taxa to infrageneric taxa and then to familial taxa (2). The Code stipulates that infraspecific taxa and infrageneric taxa that are published after 1 January 1958 must come with their nomenclatural types (2).

As of February 2022, typification information was available for a total of 147 456 taxon names, and included 124 519 collection records of type specimens (120 249 records of specimens and 4270 records of illustrations), 19 296 records of type species and 3641 records of other type taxa. Among the 10 865 currently used fungal genera, 10 038 (92.4%) had type species information. The 86 484 (57.8%) of the 149 507 fungal current species names had type specimen (specimen) information. These type specimens are preserved in a number of herbaria/fungaria and culture collections across the world, with 14.9% of specimens in the top 10 culture collections and herbaria/fungaria (Table 2). Despite these advances, the status of some types may require further clarification or even modification when the status of its associated name changes in the future. FN includes 7820 new typification records belonging to a specific genus, infrageneric genus, species or infraspecific taxon. A total of 7223 type specimens (specimens and illustrations) and 597 type species were designated separately in 1821–2022 (Figure 4B). Records of type specimens prior to 1952 are rare, with only several cases per year, but increased dramatically thereafter, particularly within the past decade. Typification for a genus or infrageneric taxa was rare until the publication of ‘*Genera of Fungi*’ in 1931. A total of 264 new type species were designated in ‘*Genera of Fungi*’. Dozens of type species were published every year after 1931.

**Table 2.** Ten agencies with the largest number of preserving fungal type specimens (specimens)

Rank	Abbreviated Name*	Full name of agency	Agency type	Country	Type specimen number* (all taxa)
1	CBS	Westerdijk Fungal Biodiversity Institute	Culture Collection	Netherlands	4591
2	IMI	CABI Genetic Resource Collection/ CABI Bioscience UK Centre	Culture Collection/Herbarium	U.K.	3276
3	MFLU	Mae Fah Luang University	Herbarium	Thailand	2079
4	PDD	Manaaki Whenua – Landcare Research	Herbarium	New Zealand	1690
5	HMAS	Fungarium (HMAS), Institute of Microbiology, Chinese Academy of Sciences	Fungarium	China	1671
6	BPI	U.S. National Fungus Collections, USDA-ARS	Fungarium	United States	1309
7	HCIO	Indian Agricultural Research Institute	Herbarium	India	1186
8	PREM	Plant Protection Research Institute	Herbarium	South Africa	960
9	HKAS	Cryptogamic Herbarium of Kunming Institute of Botany, Chinese Academy of Sciences	Herbarium	China	635
10	BRIP	Department of Agriculture and Fisheries	Culture Collection	Australia	570
	Total				17 967

*Only specimens of holotype, lectotype, neotype and epitype from parts of the data were included.

## CONCLUSIONS AND FUTURE PERSPECTIVES

**Figure 5.**
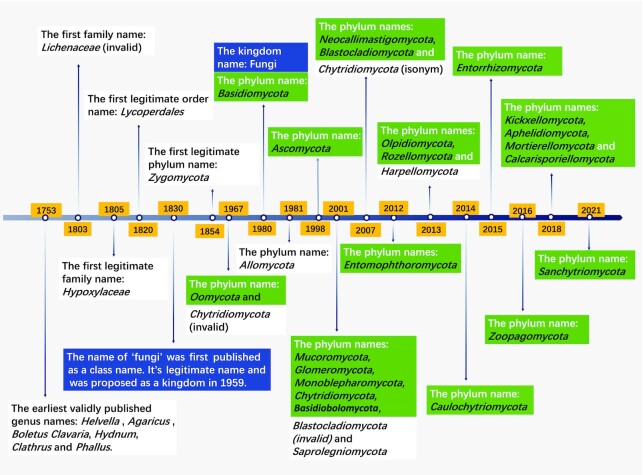
Brief history of main fungal names in fungal taxonomy (the important events in fungal taxonomy and nomenclature can be referred to the important perspective of Lücking *et al.* (1)). The taxon names in green boxes are 20 currently used phyla of kingdom fungi and other taxa of fungus-like taxa based on the research of Wijayawardene *et al.* (24,31). The taxon names in white boxes are the other phyla published in the history but not included in Wijayawardene's research and first taxa at ranks of order, family and genus published in the history. The taxon names in blue boxes are names related to kingdom name history of ‘fungi’.

FN is one of the three fungal name registration repositories recognized by the NCF and IMC. In addition to providing fast registration service for fungal name publishing, FN features a variety of functions to facilitate better access and to serve the needs of researchers all around the world, including a cross-reference database of authors, specimens, culture collections, herbaria/fungaria, publications, and various forms of illustrations and data statistics. Key historical events and advances in fungal taxonomy are also illustrated (Figure 5).

The naming of fungal species is subject to change over time (6). Along with the emergence of new sequencing methods, the identification of fungi is transforming from morphology-based to sequence-based (e.g. DNA barcode and genomic comparison). FN continues updating the system to accommodate the changing fungal nomenclature rules and collecting diverse information from multiple resources in the era of genomics.

## DATA AVAILABILITY

There are no access restrictions to academic use of the platform. Access to Fungal Names is free at https://nmdc.cn/fungalnames/. FN also provides API service to access to the database.
